# Pure platelet-rich plasma delays intervertebral disc degeneration by activating SIRT1-mediated autophagy in nucleus pulposus cells

**DOI:** 10.1186/s13018-025-06205-0

**Published:** 2025-08-22

**Authors:** Jiaheng Han, Zhili Ding, Jie Huang, Yan Zhang, Yu Ding

**Affiliations:** 1 Department of Orthopaedics, School of Medicine, South China University of Technology, Guangzhou, 51006 China; 2 Orthopedics of TCM Senior Department, The Sixth Medical Center of PLA General Hospital, Beijing, 100048 China; 3 Navy Clinical College, Fifth School of Clinical Medicine, Anhui Medical University, Hefei, China; 4 Department of Spinal Surgery, Peking University People’s Hospital, Peking University, Beijing, 100044 China

**Keywords:** Intervertebral disc degeneration, Pure platelet-rich plasma, SIRT1, Autophagy

## Abstract

**Background:**

Intervertebral disc degeneration (IVDD) is characterized by nucleus pulposus cells (NPCs) apoptosis and extracellular matrix (ECM) degradation. Impaired autophagy and mitochondrial dysfunction further accelerate disc degeneration. Pure platelet-rich plasma (P-PRP), enriched in growth factors and low in pro-inflammatory mediators, has shown regenerative potential. However, its mechanism of action, particularly the role of the autophagy-related SIRT1 pathway and mitochondrial homeostasis, remains unclear.

**Methods:**

Rabbit-derived P-PRP was prepared and analyzed for cellular content and cytokine profiling. NPCs were treated with whole blood or P-PRP, and assessed for viability (CCK-8) and migration (Transwell). An IL-1β-induced degeneration model was established, and groups were treated with SIRT1 activator (SRT1720), inhibitor (EX527), P-PRP, or P-PRP + EX527. Mitochondrial membrane potential (JC-1 staining), and apoptosis (Annexin V/PI flow cytometry) were also measured. Western blotting, immunofluorescence, qPCR, and ELISA were conducted to measure the expression of SIRT1, autophagy-related proteins, and ECM-related markers.

**Results:**

P-PRP promoted the viability and migration of NPCs, reduced apoptosis, and preserved ECM homeostasis in inflammatory conditions. P-PRP enhanced the expression of SIRT1, improved mitochondrial membrane potential, and reduced apoptosis rates. P-PRP upregulated LC3B-II and Beclin-1 expression, while downregulated p62 expression, indicating autophagy activation. EX-527 abrogated the beneficial effects of P-PRP.

**Conclusion:**

P-PRP protected against degenerative NPCs by activating functional autophagic flux and restoring mitochondrial function via the SIRT1 signaling axis. These findings provide novel mechanistic insight into PRP-based therapies and identify SIRT1 as a promising target for the treatment of IVDD.

## Introduction

Intervertebral disc degeneration (IVDD) is the leading contributor to low back pain (LBP), a highly prevalent condition characterized by significant disability and a high recurrence rate [1, 2]. Conservative approaches, including physical therapy and analgesic medication [3, 4], or surgical procedures, such as spinal fusion or discectomy [5], represent the sole management choices for IVDD in clinical practice. However, the inability of these therapies to successfully stop disc degeneration emphasizes the urgent need for biological therapies that target the underlying pathophysiological mechanisms of IVDD are needed.

The intervertebral disc (IVD) is mainly composed of the nucleus pulposus (NP), annulus fibrosus (AF), and cartilaginous endplates [2]. Of these, NP is essential to the biomechanical operation of IVD. Type II collagen (Col II) and aggrecan (ACAN) are the main constituents of the NP extracellular matrix (ECM), which resist compressive forces and maintain osmotic pressure [6]. Structural impairment of the disc is caused by disruption of ECM homeostasis, which is frequently characterized by upregulation of matrix metalloproteinases (MMPs) and a disintegrin and metalloproteinase with thrombospondin motifs (ADAMTs) [7, 8].

Platelet-rich plasma (PRP) therapy has attracted increasing attention in the treatment of musculoskeletal problems, including IVDD [9–12]. PRP comprises growth factors and cytokines that can suppress inflammation and promote tissue repair. Beyond their role as carriers of growth factors, platelets actively orchestrate tissue repair by modulating inflammation, enhancing angiogenesis, and recruiting stem cells [12, 13]. Recent studies have emphasized the multifaceted regenerative potential of platelets across musculoskeletal and other tissues [13–15]. Nevertheless, leukocytes and erythrocytes found in traditional PRP formulations can cause undesirable inflammatory reactions. In contrast, P-PRP, also called leukocyte-poor platelet-rich plasma (LP-PRP), prepared through a two-step centrifugation process, has substantially reduced cellular components and possesses better safety and efficacy profiles [16]. Recent clinical studies, using PRP for the treatment of low back pain, have reported promising but variable outcomes [17, 18], highlighting its therapeutic potential and the need for mechanistic exploration. Although P-PRP can alleviate IVDD and promote the viability of NP cells (NPCs) [10, 11], the precise molecular mechanisms underlying these protective effects remain to be elucidated.

Autophagy also plays a critical role in IVDD by preserving cell survival and homeostasis by eliminating damaged and hazardous proteins, lipids, and organelles [19, 20]. Notably, Sirtuin 1 (SIRT1), a highly conserved NAD^+^-dependent histone deacetylase, is regarded as a key regulator of autophagy [21, 22]. SIRT1 regulates multiple phases of autophagy, from initiation to degradation. The autophagy process also controls SIRT1 levels and activity [21, 22]. SIRT1 activation has been shown to postpone disc degeneration, downregulate apoptosis, and preserve extracellular matrix in IVDD [23]; however, the interplay between SIRT1-induced autophagy and disc functionality remains ambiguous.

Based on these insights, the present study aimed to investigate whether P-PRP protects against degenerative NPCs by activating SIRT1-mediated autophagy. Our findings revealed the potential of targeting the SIRT1-autophagy axis as a novel therapeutic avenue in IVDD, offering a mechanistic insight into the action of P-PRP.

## Materials and methods

### Experimental animals

NPCs and whole blood samples were independently obtained from eight healthy male New Zealand white rabbits (6 months old, 2.0–2.5 kg), provided by Beijing Baiaosi Biotechnology Co., Ltd. (license number: SYXK2021-0063). No pooling was conducted during PRP preparation or NPC isolation to ensure biological reproducibility and minimize inter-sample variability. All samples were processed individually. Cells and PRP from three randomly selected rabbits were used as independent biological replicates for key experiments. All experimental procedures were approved by the Institutional Animal Care and Use Committee of Kangtai Medical Laboratory Services Hebei Co (approval number: MDL2023-06-25-01). The study was conducted following local and national regulations and the International Association of Veterinary Editors’ consensus guidelines on animal ethics and welfare. All surgical procedures were conducted under anesthesia to minimize pain, distress, and mortality.

### Preparation and component analysis of P-PRP

P-PRP was prepared from four adult New Zealand white rabbits using a two-step centrifugation protocol. Rabbits were anesthetized with 2% isoflurane in oxygen using a precision vaporizer. Arterial blood was drawn via cardiac puncture using citrate-containing tubes. Five milliliters of whole blood were used for routine blood tests, while the remaining blood was gently mixed and centrifuged at 1200 × g for 10 min. The resulting three layers included plasma (upper), red blood cells (lower), and a buffy coat in between. The upper part of the buffy coat was discarded, and the supernatant was transferred and centrifuged at 1000 × g for 10 min. The lower portion of the second supernatant was collected and designated as P-PRP. Leukocyte and platelet concentrations in P-PRP were measured using a blood analyzer.

### Isolation and culture of rabbit NPCs

NPCs were harvested from four healthy 6-month-old rabbits. After euthanasia under anesthesia, lumbar IVDs were rapidly dissected under sterile conditions. The annulus fibrosus was removed, and the NP tissue was harvested using a stereomicroscope to prevent contamination. NP tissues were finely minced and digested in 0.2 mg/mL type II collagenase (Solarbio, Beijing, CN) in DMEM/F12 medium (Gibco) at 37 °C for 1 h with gentle agitation. After digestion, the cells were centrifuged at 1500 rpm for 10 min, resuspended in high-glucose DMEM, and seeded at a density of 1 × 10^6^ cells/mL. The cells were cultured at 37 °C in 5% CO_2_, and the medium was changed every 2 days. Third-passage NPCs were used for subsequent experiments. The procedure followed the minimum reporting standards for NP cell culture as outlined by the ORS spine Sect. [24].

### Establishment of degeneration model and experimental grouping

The obtained NPCs were seeded in 6-well plates and divided into six experimental groups. All groups were cultured for a total of 48 h. The experimental conditions were as follows: (1) the control group, which was treated with complete medium (DMEM/F12: fetal bovine serum = 9:1) for 48 h without any stimulation; (2) the IVDD group, which was treated with IL-1β (10 ng/mL, MedChemExpress, HY-P73150, USA) for 48 h; (3) the SRT1720 group, which was treated with IL-1β (10 ng/mL) for 24 h, followed by co-incubation with IL-1β (10 ng/mL) and SRT1720 (1 µmol/L, MedChemExpress, HY-10532, USA), a selective SIRT1 activator, for an additional 24 h; (4) the EX527 group, which was treated as in the SRT1720 group but with the addition of EX-527 (5 µmol/L, MedChemExpress, HY-15452, USA), a selective SIRT1 inhibitor, during the second 24 h period; (5) the PRP group, which was treated with IL-1β (10 ng/mL) for 24 h, then co-incubated with IL-1β (10 ng/mL) and 200 µL of P-PRP for another 24 h, achieving a final PRP concentration of 10% (v/v); and (6) the PRP + EX527 group, which was treated identically to the PRP group, with the addition of EX-527 (5 µmol/L) during the final 24 h.

### Mitochondrial membrane potential (ΔΨm) assay

NPCs were seeded in 6-well plates and treated as described previously. The cells were washed three times with PBS and dark incubated with 2 µM JC-1 dye (Beyotime, C2006, CN) at 37 °C for 20 min. Healthy cells showed red fluorescence (J-aggregates, 590 nm), while apoptotic cells showed green fluorescence (monomers, 527 nm). After washing, fluorescence was observed under a microscope and quantified using a microplate reader at 530 and 590 nm. The red/green fluorescence ratio was calculated to assess mitochondrial function.

### Immunofluorescence staining

After reaching 90% confluence, NPCs were placed into 24-well plates. Following washing with PBS, the samples were treated with 4% paraformaldehyde at 25 °C for 15 min and washed three times with PBS. The samples were permeabilized by exposure to 0.5% Triton-X 100 at 25 °C for 15 min. After washing the cells with PBS, the primary antibodies against LC3B (ThermoFisher, PA1-16930, USA) were incubated with TBST at 4 °C overnight. After washing, the cells were exposed to a fluorescent secondary antibody and incubated at 37 °C for 2 h. Cell nuclei were stained with DAPI at room temperature for 5 min, followed by additional PBS washes. A nonfluorescent quencher was slowly added before placing the slides under glass covers. The images were observed under a fluorescence microscope (Leica, DM3000, DE).

### Annexin V-FITC/PI staining for apoptosis

NPCs were collected, washed with cold PBS, and resuspended in 1× Annexin V binding buffer (1 × 10⁶/mL; 4 A BIOTECH, FXP018, CN). A 100 µL aliquot was dark incubated with 5 µL of Annexin V-FITC (4 A BIOTECH, FXP018, CN) and 5 µL of PI (4 A BIOTECH, FXP018, CN) for at room temperature 15 min. After adding 400 µL of buffer, the samples were analyzed by flow cytometry. The cells were divided into four subpopulations based on fluorescence staining and their position on the dot plot: viable cells located in the lower left quadrant (Annexin V⁻/PI⁻), early apoptotic cells in the lower right quadrant (Annexin V⁺/PI⁻), late apoptotic or secondary necrotic cells in the upper right quadrant (Annexin V⁺/PI⁺), and necrotic cells in the upper left quadrant (Annexin V⁻/PI⁺). The proportions of early and late apoptotic cells were quantified and summed to calculate the total apoptotic rate for statistical comparison.

### ELISA for cytokine quantification

The concentrations of inflammatory cytokines in P-PRP preparations and extracellular matrix-related proteins in NPC culture supernatants were measured using ELISA kits following the manufacturers’ instructions. Specifically, the levels of TNF-α (MDL, MD12311, CN) and IL-1β (MDL, MD15478, CN) were measured P-PRP samples, while PDGF-A (MDL, MD14587, CN), TGF-β1 (MDL, MD12352, CN), MMP3 (MDL, MD15632, CN), ADAMTS4 (MDL, MD15747, CN), and ADAMTS5 (MDL, MD15749, CN) expression were quantified after treatment. Briefly, 96-well plates were coated with monoclonal antibodies at 4 °C overnight, blocked with 2% BSA for 1 h, and incubated with samples for 60–120 min. After washing, HRP-conjugated secondary antibodies and TMB substrate were sequentially added. The reaction was terminated using a stop solution, and absorbance was read at 450 nm using a microplate reader (Bio-Rad, Model 550, USA).

### Western blotting

NPCs were lysed using RIPA buffer with protease/phosphatase inhibitors. Total protein was quantified using the BCA (MDL, MD913053, CN) method, separated by SDS-PAGE (Bio-Rad, USA), and transferred onto PVDF membranes (Millipore, Billerica, USA). The membranes were blocked with 5% skimmed milk in TBST (Beyotime) at room temperature for 2 h and incubated with primary anti-bodies against β-actin (1:1000, MDL, MD6553, CN), LC3B (1:1500, ThermoFisher, PA1-16930, USA), P62/SQSTM1 (1:1000, Proteintech, 18420-1-AP, USA), Beclin-1 (1:500, Proteintech, 11306-1-AP, USA), Aggrecan (1:1000, Proteintech, 13880-1-AP, USA), Collagen II (1:1000, Bioss, bs-10589R, CN) and SIRT1 (1:1000, Bioss, bs-2257R, CN). After washing with TBST, the membrane was incubated with HRP-conjugated goat anti-rabbit IgG (1:1000, ZEN BIO, 511103, CN) at room temperature for 1 h. An enhanced chemiluminescence reagent (UVP, GelDoc-It310, USA) was utilized for protein identification. A chemiluminescence imaging system (Bio-Rad, 170–8280, USA) was employed for imaging.

### Real-time quantitative polymerase chain reaction (RT-qPCR)

Total RNA was extracted from NPCs using TRIzol and reverse-transcribed into cDNA. mRNA levels were measured using SYBR Green-based RT-qPCR. Relative gene expression level was calculated using the 2^-ΔΔ*Ct*^ method, considering β-actin as the internal control. The sequence of genes primarily measured in this study is shown in Table 1.

**Table 1:** Primers used for gene expression analysis

Gene		Primer Sequence
*Actin for rabbit*	F	ACGACATGGAGAAGATCTGGCAC
	R	AACGTCTCGAACATGATCTGGGT
*LC3B for rabbit*	F	TCAAAATCATCAGGAGGCGC
	R	ACCATGTACAGGAAGCCGTC
*p62 for rabbit*	F	TCGCCTTCTCCAGTGATGAG
	R	CCGGCACTCCTTCTTCTCTT
*Beclin1 for rabbit*	F	AACGAGGATGACAGTGAGCA
	R	GCGGTTCTTTTCCACGTCTT
*Mmp3 for rabbit*	F	ACTTCAGTACCTTCCCTGGC
	R	AATGGCAGCATCAACAGCAT
*Acan for rabbit*	F	TTTTGGAACTCAGCGGTGTG
	R	CTAAAATACGGGGTGCGTGG
*Col2a1 for rabbit*	F	GATAGACCCCAACCAAGGCT
	R	CACCAGTTCTTCTTGGGCAC

### Cell viability assay (CCK-8)

Cell viability was measured using the cell counting kit-8 (CCK-8; Dojindo, CK04, JP) following the manufacturer’s instructions. NPCs were seeded into 96-well plates at a density of 3,000 cells per well in 100 µL of complete medium. After incubation for 12 h to allow cell attachment, the cells were treated with experimental reagents based on the designed groupings. At the indicated time points, 10 µL of the CCK-8 solution was added to each well, followed by incubation at 37 °C for 1 h. The absorbance was read at 450 nm using a microplate reader (Bio-Rad, Model 550, USA).

### Transwell assay

NPC migration was detected in a Transwell chamber (8.0 μm, 24-well, Corning, USA). NPCs were trypsinized at 80–90% confluence, washed with PBS and serum-free medium, counted, and resuspended to a final density of 2 × 10⁵ cells/mL. For each insert, 100–150 µL of the cell suspension was added to the upper chamber, while the lower chamber was filled with 600–800 µL of the complete medium containing 10% FBS as a chemoattractant. The chambers were incubated at 37 °C for 48 h. After incubation, non-migrated cells on the upper membrane surface were removed using a cotton swab. The inserts were fixed in 4% paraformaldehyde (XiLONG SCIENTIFIC, 1710091, CN) at room temperature for 30 min and stained with crystal violet (MDL, MD911626, CN) for 1–5 min. Excess dye was removed by rinsing in PBS (MDL, MD911702, CN). Membranes were gently washed, and non-migrated cells were removed from the upper surface. The membranes were then excised using a scalpel, placed onto glass slides with the bottom side facing up, and sealed with neutral resin (Gibco, 25200-072, USA). Migrated cells were counted under a light microscope (NOVEL, XS-2100, CN) in at least five random fields per insert.

### Statistical analysis

Statistical analyses were conducted using SPSS 22.0 (IBM Corp., USA). GraphPad Prism 9.0 (GraphPad Software Inc., CA) was used for data visualization. One-way analysis of variance (ANOVA) followed by Tukey’s post hoc test was employed for multiple group comparisons. Two-group comparisons were conducted using unpaired t-tests. A value of *P* < 0.05 was considered statistically significant. Significance levels are indicated as follows: *P < 0.05, **P < 0.01, ***P < 0.001, ****P < 0.0001; ns indicates no statistical significance.

## Results

### Characterization of P-PRP and its effects on nucleus pulposus cells

**Fig. 1:**
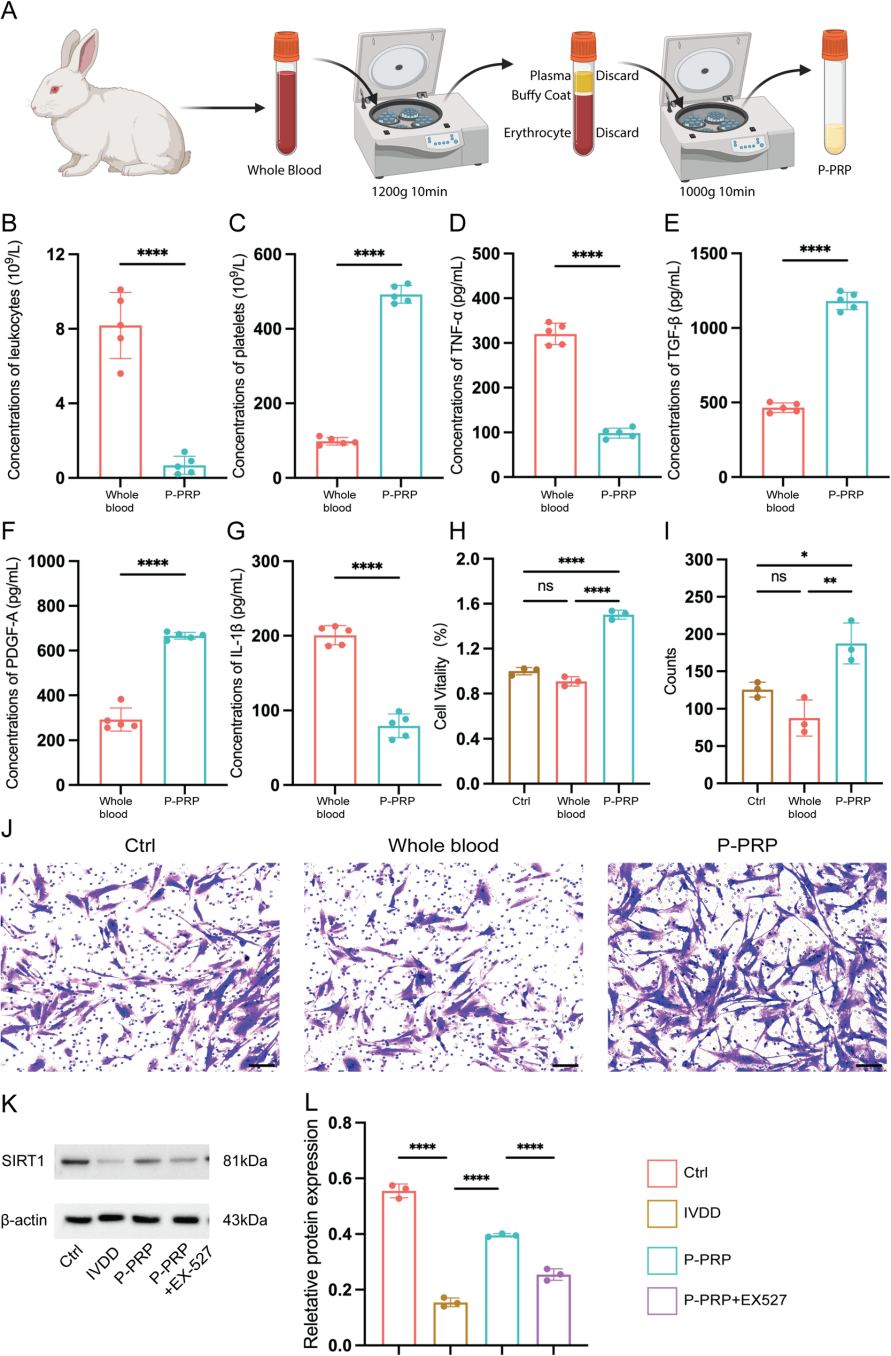
Characterization of P-PRP and its effects on inflammatory NPCs. **A**, Diagram of P-PRP preparation. **B**-**C**, Leukocyte and platelet concentration. **D**-**G**, ELISA results show the levels of TNF-α, TGF-β, PDGF-A, and IL-1β. **H**, CCK-8 assay result show cell viability. **I**-**J**, Transwell assay evaluate the migration ability of NPCs after different treatments (Bar = 100 μm). **K**-**L**, the representative WB images (**K**) quantification data (**L**) of SIRT1 protein levels in NPCs under different treatments. Data are shown as mean ± SD; *P < 0.05, **P < 0.01, ***P < 0.001, ****P < 0.0001. ns, no significance

We used rabbits to extract blood, prepare P-PRP, and determine its quality and composition. The preparation process is described in Fig. 1A. In whole blood, the mean platelet concentration was 98.2 ± 9.8 × 10⁹/L, and leukocyte concentration was 8.2 ± 1.8 × 10⁹/L (Fig. 1B and C). P-PRP exhibited a marked increase in platelet concentration (492.4 ± 23.6 × 10⁹/L), along with a substantial reduction in leukocyte content (0.7 ± 0.5 × 10⁹/L). The obtained P-PRP exhibited effective platelet enrichment and leukocyte depletion, consistent with the characteristics of LP-PRP. Furthermore, ELISA revealed that P-PRP contained significantly higher levels of growth factors, including transforming growth factor-beta (TGF-β: 1181 ± 58.57 pg/mL) and platelet-derived growth factor-A (PDGF-A: 666.2 ± 15.25 pg/mL), compared to whole blood (TGF-β: 465.2 ± 31.79 pg/mL; PDGF-A: 292.8 ± 51.82 pg/mL; *P* < 0.0001 for both; Fig. 1E and F). Notably, the concentrations of pro-inflammatory cytokines were significantly lower in P-PRP, with TNF-α reduced from 320.4 ± 23.83 pg/mL to 98.4 ± 11.01 pg/mL (*P* < 0.001) and IL-1β from 200.7 ± 12.79 pg/mL to 79.39 ± 15.86 pg/mL (*P* < 0.0001), suggesting an anti-inflammatory profile (Fig. 1D and G). These results suggest that the P-PRP component, which includes growth factors and, to a lesser extent, pro-inflammatory factors, can decelerate disc degeneration and decrease the risks [25–27]. To explore the potential biological effects of P-PRP in IVDD, we treated IL-1β-stimulated NPCs with either whole blood or P-PRP. CCK-8 assay revealed that P-PRP significantly enhanced the viability of degenerative NPCs compared to whole blood, with absorbance values increasing from 0.90 ± 0.04 (whole blood group) to 1.5 ± 0.04 (P-PRP group, *P* < 0.0001; Fig. 1H). Moreover, Transwell migration assays indicated that P-PRP promoted cellular migration in compared to whole blood. These data suggest that P-PRP can restore the functions of NPCs in degenerative conditions (Fig. 1I-J). We next measured the expression of SIRT1 to investigate the underlying mechanisms. Western blotting revealed that the protein levels of SIRT1 were reduced in IL-1β-induced degenerative NPCs compared to the control group. Interestingly, treatment with P-PRP restored SIRT1 expression, whereas co-treatment with EX-527 effectively reversed this effect (Fig. 1K-L). These results suggest that P-PRP can enhance NPC viability at least partly through the reactivation of SIRT1 signaling, laying the foundation for its downstream effects on autophagy and matrix homeostasis.

### P-PRP activated SIRT1 to alleviate matrix degradation in degenerative NPCs

**Fig. 2:**
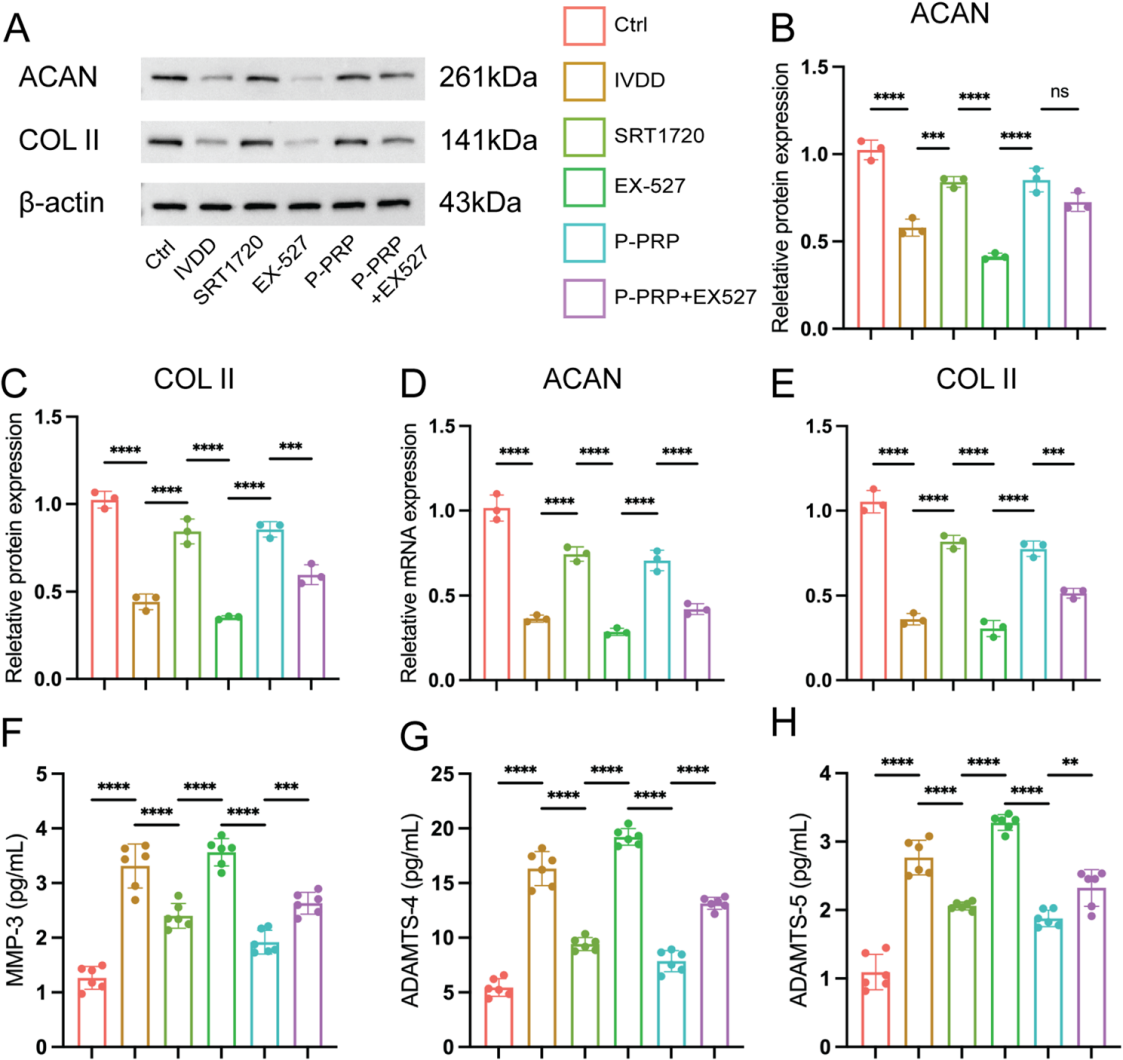
P-PRP alleviates ECM degradation via SIRT1 activation in degenerative NPCs. **A**-**C**, the representative WB images (**A**) quantification data (**B**-**C**) of ACAN and COL II protein levels in NPCs under different treatments. **D**-**E**, the gene expression of ACAN and COL II was analyzed by RT-qPCR. **F**-**H**, ELISA analysis show secretion of MMP-3, ADAMTS-4 and ADAMTS-5. Data are shown as mean ± SD; *P < 0.05, **P < 0.01, ***P < 0.001, ****P < 0.0001. ns, no significance

We measured ECM-related proteins and the expression of various catabolic mediators under different treatments to explore the specific mechanisms by which P-PRP improves ECM degradation. Western blotting showed that IL-1β stimulation significantly downregulated the expression of ACAN (0.57 ± 0.04) and COL II (0.44 ± 0.04) in NPCs compared to the control group (ACAN: 1.00 ± 0.05; COL II: 1.00 ± 0.08; *P* < 0.001 for all). These alterations were reversed by SRT1720 (ACAN: 0.84 ± 0.03; COL II: 0.84 ± 0.07) and P-PRP (ACAN: 0.85 ± 0.07; COL II: 0.85 ± 0.04), whereas EX-527 further exacerbated matrix degradation. Notably, the protective effect of P-PRP on matrix integrity was attenuated after co-administering EX-527 (Fig. 2A-C). RT-qPCR showed consistent regulation of ACAN and COL II at the mRNA level (Fig. 2D-E). Furthermore, ELISA revealed that IL-1β stimulation significantly upregulated the secretion of matrix-degrading enzymes, including MMP-3 (3.31 ± 0.4 pg/mL), ADAMTS-4 (16.32 ± 1.56 pg/mL), and ADAMTS-5 (2.77 ± 0.25 pg/mL), compared to the control group (MMP-3: 1.26 ± 0.2 pg/mL; ADAMTS-4: 5.44 ± 0.81 pg/mL; ADAMTS-5: 1.1 ± 0.25 pg/mL; *P* < 0.0001 for all). These increases suggest an accelerated degradation of the aggrecan-rich matrix and potential structural disruption [28, 29]. MMP-3, ADAMTS-4, and ADAMTS-5 secretion was markedly suppressed by SRT1720 and P-PRP. For example, MMP-3 levels were reduced from 3.31 ± 0.4 pg/mL in the IL-1β group to 1.91 ± 0.2 pg/mL in the P-PRP group (*P* < 0.0001), and similar trends were observed for ADAMTS-4 (16.32 ± 1.56 vs. 7.85 ± 0.97 pg/mL) and ADAMTS-5 (2.77 ± 0.25 vs. 1.88 ± 0.12 pg/mL; *P* < 0.0001 for both; Fig. 2F-H). The protective effects of SRT1720 and P-PRP were reversed after inhibiting SIRT1 (Fig. 2H-J). These data suggest that SIRT1 plays a key role in the therapeutic effects of P-PRP on IVDD by protecting ECM integrity.

### P-PRP activated autophagy through SIRT1 signaling in degenerative NPCs

**Fig. 3:**
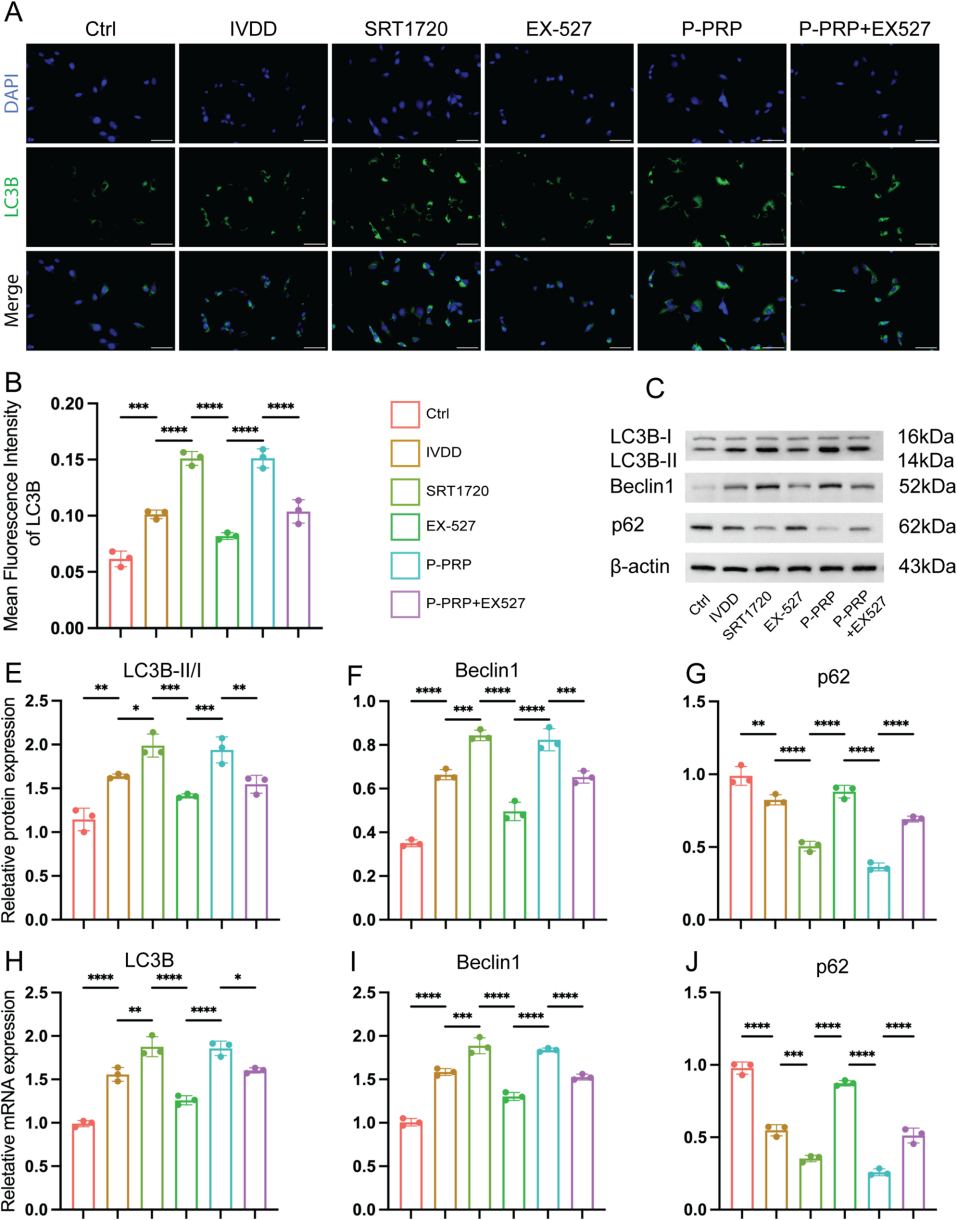
P-PRP activates autophagy in NPCs through SIRT1 signaling. **A**-**B**, representative images of Immunofluorescence staining of LC3B (**A**) and quantification of LC3B (**B**) in NPCs under different treatments (Bar = 100 μm). C-G, the representative WB images (**C**) quantification data (**E**-**G**) of the LC3B-II/I ratio, SQSTM1/p62, and Beclin-1 protein levels. **H**-**J**, the gene expression of LC3B, SQSTM1/p62, and Beclin-1 was analyzed by RT-qPCR. Data are shown as mean ± SD; *P < 0.05, **P < 0.01, ***P < 0.001, ****P < 0.0001. ns, no significance

Since SIRT1 regulates autophagy, we next assessed whether P-PRP exerts its protective effects by activating autophagy. Immunofluorescence staining revealed increased levels of microtubule-associated protein 1 light chain 3 (LC3B), a key marker of autophagosomes, in SRT1720-treated and P-PRP-treated cells, whereas EX-527 reduced the fluorescence intensity of LC3B (Fig. 3A and B). Next, we measured the protein expression of LC3B, Bcl-2-interacting protein 1 (Beclin-1), a crucial autophagy initiator, and SQSTM1/p62 (sequestosome 1), an established marker of autophagic clearance, in NPCs. Western blotting showed that SRT1720 and P-PRP enhanced the LC3B-II/I ratio and Beclin-1 expression and decreased SQSTM1/p62 levels, suggesting active degradation of autophagic cargo and restoration of the autophagic flux [30]. However, EX-527 reversed these effects (Fig. 3C-G). RT-qPCR revealed consistent patterns, showing that LC3B and Beclin-1 mRNA levels were upregulated and SQSTM1/p62 levels were downregulated in the SRT1720 and P-PRP groups (Fig. 3H-J). EX-527 reversed these alterations (Fig. 3H-J). Although the IL-1β-induced degenerative model exhibited elevated Beclin-1 expression, this likely reflects a compensatory response to stress rather than effective autophagy. These findings suggest that P-PRP protects degenerative NPCs by restoring functional autophagy, and this effect critically relies on SIRT1 signaling.

### P-PRP protects NPCs against degeneration by improving mitochondrial function and reducing apoptosis

**Fig. 4:**
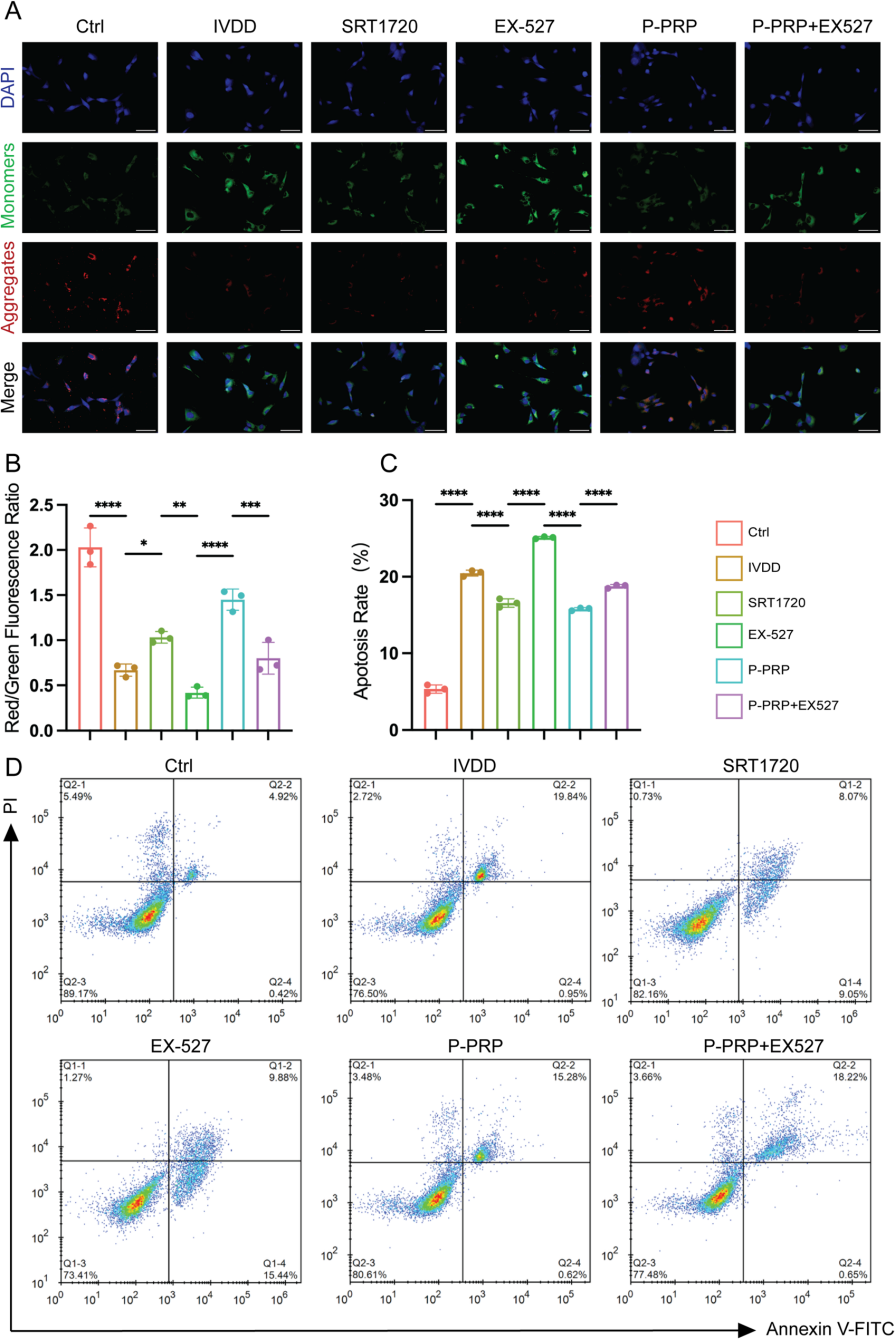
P-PRP preserves mitochondrial membrane potential and reduces apoptosis via SIRT1. **A**-**B**, JC-1 staining and quantification of red/green fluorescence ratio show mitochondrial membrane potential (Bar = 100 μm). **C**-**D**, Flow cytometry analysis show apoptosis rates in NPCs following different treatment. Viable cells are Annexin V^-^/PI^-^ (lower left quadrant), early apoptotic cells are Annexin V⁺/PI⁻ (lower right), late apoptotic or secondary necrotic cells are Annexin V⁺/PI⁺ (upper right), and necrotic cells are Annexin V^-^/PI^+^ (upper left). Data are shown as mean ± SD; *P < 0.05, **P < 0.01, ***P < 0.001, ****P < 0.0001. ns, no significance

We measured apoptosis and mitochondrial function in degenerative NPCs to better understand the protective mechanisms of P-PRP. JC-1 staining showed that both SRT1720 and P-PRP successfully reversed IL-1β-induced mitochondrial depolarization (Fig. 4A and B). However, co-treatment with EX-527 reduced the positive effects of P-PRP, indicating that SIRT1 is involved in the protective effects of P-PRP on mitochondrial membrane potential (Fig. 4A and B). Moreover, flow cytometry showed that IL-1β-stimulated NPCs significantly enhanced the apoptosis rate of NPCs (20.47% ± 0.38%) compared to the control group (5.34% ± 0.52%, *P* < 0.0001), while SRT1720 (16.57% ± 0.55%) and P-PRP (15.77% ± 0.19%) had the opposite effect (*P* < 0.001 for both vs. IL-1β). Additionally, EX-527 inhibited the anti-apoptotic effect of P-PRP (Fig. 4C and D). These results collectively suggest that P-PRP protects against IVDD by maintaining mitochondrial function and preventing apoptosis via SIRT1.

## Discussion

By activating the SIRT1/autophagy signaling axis, we showed that P-PRP, a platelet-rich and growth factor-rich plasma preparation with substantially reduced leukocyte and erythrocyte content, protects against degenerative NPCs. These results suggest that P-PRP reduces IVDD by coordinating changes in matrix metabolism, inflammation, and mitochondrial integrity via SIRT1.

P-PRP exhibited a positive cytokine profile with lower levels of TNF-α and IL-1β, two important factors involved in the pathogenesis of IVDD [7, 31]. In the meantime, by promoting ECM synthesis, improving NPC survival, and regulating inflammatory responses, enrichment in regenerative growth factors (TGF-β and PDGF-A) protects against IVDD [25, 32, 33]. P-PRP provides better safety and therapeutic efficacy than traditional PRP preparations, which may cause unfavorable immune reactions because of their leukocyte content. Next, we confirmed that P-PRP promotes NPC viability and migration in degenerative environments.

Notably, our data provide direct evidence showing that P-PRP restores the expression of SIRT1, a NAD⁺-dependent deacetylase that regulates autophagy and mitochondrial function [34]. SIRT1 expression was downregulated in IL-1β-induced degenerative NPCs but upregulated after treatment with P-PRP. Importantly, this effect was abrogated by EX-527, confirming the specificity of this regulatory axis. These findings confirm that P-PRP reactivates the SIRT1 signaling, laying the foundation for downstream autophagy regulation and phenotypic preservation. Furthermore, Western blotting and RT-qPCR revealed increased expression levels of ACAN and COL II, showing that these functional enhancements were accompanied by the restoration of ECM homeostasis. Furthermore, ELISA showed that P-PRP significantly reversed the increase in the secretion of MMP-3, ADAMTS-4, and ADAMTS-5 in the matrix of degenerative NPCs, which causes disc structural failure of the disc [35, 36]. Although the regenerative potential of PRP has been reported in previous studies, little is known about the mechanisms underlying these effects; our study fills this important knowledge gap. The protective effects of P-PRP on ECM metabolism and NPC phenotype were significantly reduced after SIRT1 blockade, underscoring its significance as a downstream effector. In addition to improving our knowledge regarding the effects of P-PRP, this study identified SIRT1 as a crucial regulatory target for the delayed effects of R-PRP on IVDD.

Moreover, SIRT1 activation enhanced the autophagic flux, which plays a pivotal role in the maintenance of NPC viability under degenerative stress. Although autophagy-related markers were elevated in the IVDD model, this upregulation may reflect a compensatory but potentially insufficient response to cellular stress rather than fully functional autophagy. Previous studies have suggested that accumulation of autophagy markers in degenerative contexts may indicate impaired autophagosome clearance or abnormal autophagic flux rather than productive autophagy [37]. To further validate this interpretation, future studies should incorporate dynamic autophagy flux assays, such as tandem mCherry-GFP-LC3 reporters, or employ phase-specific pharmacological inhibitors like Bafilomycin A1 or chloroquine, to distinguish between autophagosome formation and degradation. These approaches would help elucidate the functional relevance of SIRT1 in the regulation of autophagy under degenerative conditions, and clarify whether the observed changes in autophagy-related markers reflect true alterations in autophagic flux and their potential impact on disc cell function. SIRT1 activation by P-PRP or the pharmacologic agonist SRT1720 [38, 39] elevated LC3B-II and Beclin-1 expression and reduced SQSTM1/p62 accumulation. SQSTM1/p62 is a selective substrate that is degraded during autophagy, while LC3B and Beclin-1 are necessary for autophagosomes formation [40–42]. Reduced levels of LC3B and Beclin-1 and SQSTM1/p62 accumulation in degenerative NPCs suggest compromised autophagic activity, which enhances inflammation, ECM degradation, and cell death [40–42]. Conversely, EX-527 reversed these effects, especially in the P-PRP + EX-527 group, suggesting the crucial function of SIRT1 in P-PRP-induced autophagy.

In addition to autophagy, P-PRP inhibited apoptosis in degenerative NPCs and maintained mitochondrial membrane potential, a crucial measure of mitochondrial function and cellular energy status. Decreased MMP levels in IVDD suggest mitochondrial dysfunction, which accelerates disc degeneration by inducing NPC death and ECM degradation [42–44]. Our findings imply that P-PRP may maintain mitochondrial function through SIRT1-dependent autophagy, as SIRT1 also controls mitochondrial function through mitophagy. P-PRP preserves the integrity of NPCs under degenerative stress through a comprehensive cytoprotective mechanism that is highlighted by dual regulation of apoptotic and metabolic pathways.

Together, our results revealed an unexplored mechanism by which P-PRP activates autophagic flux, restores the integrity of ECM, and maintains mitochondrial function through SIRT1 activation. These findings offer a mechanistic insight into the application of P-PRP as a regenerative treatment for IVDD. Notably, by identifying a specific intracellular signaling cascade, this work expands the therapeutic potential of PRP beyond its ability to provide growth factors.

The regenerative effects of PRP are primarily attributed to its platelets, which play a central role in coordinating tissue healing. Platelets release various bioactive molecules that regulate inflammation, matrix remodeling, and cell survival in damaged tissues [13, 14]. Accumulating evidence suggests that PRP exerts anti-inflammatory, anti-apoptotic, and pro-anabolic effects on disc cells via several pathways, including NF-κB inhibition, PI3K/Akt activation, and enhanced matrix synthesis [45, 46]. However, variability in PRP composition and delivery protocols contributes to inconsistent outcomes across studies. Given its defined leukocyte-poor profile, P-PRP represents a promising formulation for disc regeneration. Future in vivo studies should explore its optimal dosing, injection frequency, and potential combination with biomaterials to prolong its retention in the disc and enhance its therapeutic efficacy.

However, this study had several limitations. First, all experiments were conducted in vitro using an IL-1β-induced degeneration model, which reproduced the inflammatory response in IVDD but did not fully reflect its multifaceted pathogenesis. Second, cell culture was conducted under normoxic conditions (21% O₂), whereas NPCs reside in a hypoxic niche (1–5% O₂) in vivo. Given that hypoxia may enhance SIRT1-mediated autophagy, future studies under physiological oxygen levels are needed to better simulate the native microenvironment. Third, rabbit intervertebral discs differ from human discs in terms of size, biomechanics, and cellular composition. For instance, the persistence of notochordal cells in rabbit intervertebral discs may affect their responsiveness to regenerative stimuli [47]. Future studies using large animal models, such as porcine or bovine, may improve translational relevance. Fourth, although autophagy activation was supported by increased LC3B-II/I ratio and decreased P62 protein levels, the lack of lysosomal inhibitors limits definitive assessment of autophagic flux. Finally, although P-PRP activated SIRT1, the specific component(s) responsible for SIRT1 activation remain to be identified.

In conclusion, our study showed that P-PRP activates SIRT1-mediated autophagy, stabilizes the mitochondria, restores the ECM, and strongly protects against degenerative NPCs. These results offer strong preclinical evidence in favor of continued development of P-PRP as a secure, autologous biological approach to IVD regeneration.

## Data Availability

No datasets were generated or analysed during the current study.

## Abbreviations

IVDD: Intervertebral disc degeneration
NP: Nucleus pulposus
AF: Annulus fibrosus
NPCs: Nucleus pulposus cells
SIRT1: Sirtuin 1
P-PRP: Pure platelet-rich plasma
LC3B: Microtubule-associated protein 1 light chain 3 beta
Beclin-1: Bcl-2-interacting protein 1
ADAMTS: A disintegrin and metalloproteinase with thrombospondin motifs
MMP: Matrix metalloproteinase
TNF-α: Tumor necrosis factor-α
TGF-β: Transforming growth factor-beta
IL-1β: Interleukin-1β
ACAN: Aggrecan
COL II: Type II collagen

## References

[1] Global regional, national burden of low back pain. 1990–2020, its attributable risk factors, and projections to 2050: a systematic analysis of the global burden of disease study 2021. Lancet Rheumatol. 2023;5(6):e316–29. 37273833 10.1016/S2665-9913(23)00098-X PMC10234592

[2] Diwan AD, Melrose J. Intervertebral disc degeneration and how it leads to low back pain. JOR Spine. 2023;6(1):e1231. 36994466 10.1002/jsp2.1231 PMC10041390

[3] O’Keeffe M, George SZ, O’Sullivan PB, O’Sullivan K. Psychosocial factors in low back pain: letting go of our misconceptions can help management. Br J Sports Med. 2019;53(13):793–4. 30154205 10.1136/bjsports-2018-099816

[4] Silva-Correia J, Correia SI, Oliveira JM, Reis RL. Tissue engineering strategies applied in the regeneration of the human intervertebral disk. Biotechnol Adv. 2013;31(8):1514–31. 23911974 10.1016/j.biotechadv.2013.07.010

[5] Mochida J, Sakai D, Nakamura Y, Watanabe T, Yamamoto Y, Kato S. Intervertebral disc repair with activated nucleus pulposus cell transplantation: a three-year, prospective clinical study of its safety. Eur Cell Mater. 2015;29:202–12. discussion 212. 25794529 10.22203/ecm.v029a15

[6] Risbud MV, Shapiro IM. Role of cytokines in intervertebral disc degeneration: pain and disc content. Nat Rev Rheumatol. 2014;10(1):44–56. 24166242 10.1038/nrrheum.2013.160 PMC4151534

[7] Wang Y, Che M, Xin J, Zheng Z, Li J, Zhang S. The role of IL-1β and TNF-α in intervertebral disc degeneration. Biomed Pharmacother. 2020;131:110660. 32853910 10.1016/j.biopha.2020.110660

[8] Wen P, Zheng B, Zhang B, Ma T, Hao L, Zhang Y. The role of ageing and oxidative stress in intervertebral disc degeneration. Front Mol Biosci. 2022;9:1052878. 36419928 10.3389/fmolb.2022.1052878 PMC9676652

[9] Sezen GB, Boyalı O, Karip B, Aktaş S, Savrunlu EC, Chasan M, Kaplan N, Civelek E, Kabataş S. Retrospective clinical and radiological comparison of intradiscal Ozone and Ozone + PRP therapy results in patients with intervertebral disc degeneration. Turk J Anaesthesiol Reanim 2025. 10.4274/TJAR.2025.24183. Epub ahead of print. 10.4274/TJAR.2025.241831 PMC12519906 40366116

[10] Kawabata S, Akeda K, Yamada J, Takegami N, Fujiwara T, Fujita N, Sudo A. Advances in Platelet-Rich plasma treatment for spinal diseases: A systematic review. Int J Mol Sci. 2023;24(8). 10.3390/ijms24087677 PMC10145581 37108837

[11] Wang SZ, Chang Q, Lu J, Wang C. Growth factors and platelet-rich plasma: promising biological strategies for early intervertebral disc degeneration. Int Orthop. 2015;39(5):927–34. 25653173 10.1007/s00264-014-2664-8

[12] Gupta A, Jeyaraman M, Maffulli N. Common medications which should be stopped prior to Platelet-Rich plasma injection. Biomedicines. 2022;10(9). 10.3390/biomedicines10092134 PMC9495905 36140235

[13] Andia I, Maffulli N. Blood-Derived products for tissue repair/regeneration. Int J Mol Sci. 2019;20(18). 10.3390/ijms20184581 PMC6770158 31533202

[14] Andia I, Maffulli N. A contemporary view of platelet-rich plasma therapies: moving toward refined clinical protocols and precise indications. Regen Med. 2018;13(6):717–28. 30246605 10.2217/rme-2018-0042

[15] Andia I, Maffulli N. Some patients (and some of us) respond better to some biological therapies: the as yet unsolved conundrum. J Orthop Traumatol. 2018;19(1):1. 30128775 10.1186/s10195-018-0505-z PMC6102158

[16] Dohan Ehrenfest DM, Rasmusson L, Albrektsson T. Classification of platelet concentrates: from pure platelet-rich plasma (P-PRP) to leucocyte- and platelet-rich fibrin (L-PRF). Trends Biotechnol. 2009;27(3):158–67. 19187989 10.1016/j.tibtech.2008.11.009

[17] Schol J, Tamagawa S, Volleman TNE, Ishijima M, Sakai D. A comprehensive review of cell transplantation and platelet-rich plasma therapy for the treatment of disc degeneration-related back and neck pain: A systematic evidence-based analysis. JOR Spine. 2024;7(2):e1348. 38919468 10.1002/jsp2.1348 PMC11196836

[18] Lee JS, Lee SB, Kang KY, Oh SH, Chae DS. Review of recent treatment strategies for lumbar disc herniation (LDH) focusing on nonsurgical and regenerative therapies. J Clin Med. 2025;14(4). 10.3390/jcm14041196 PMC11856164 40004728

[19] Wang J, Zhang Y, Cao J, Wang Y, Anwar N, Zhang Z, Zhang D, Ma Y, Xiao Y, Xiao L, et al. The role of autophagy in bone metabolism and clinical significance. Autophagy. 2023;19(9):2409–27. 36858962 10.1080/15548627.2023.2186112 PMC10392742

[20] Cheng Z, Gan W, Xiang Q, Zhao K, Gao H, Chen Y, Shi P, Zhang A, Li G, Song Y, et al. Impaired degradation of PLCG1 by chaperone-mediated autophagy promotes cellular senescence and intervertebral disc degeneration. Autophagy. 2025;21(2):352–73. 39212196 10.1080/15548627.2024.2395797 PMC11759519

[21] Yang Y, Liu Y, Wang Y, Chao Y, Zhang J, Jia Y, Tie J, Hu D. Regulation of SIRT1 and its roles in inflammation. Front Immunol. 2022;13:831168. 35359990 10.3389/fimmu.2022.831168 PMC8962665

[22] Chen C, Zhou M, Ge Y, Wang X. SIRT1 and aging related signaling pathways. Mech Ageing Dev. 2020;187:111215. 32084459 10.1016/j.mad.2020.111215

[23] Zhang Z, Wu J, Teng C, Wang J, Yu J, Jin C, Wang L, Wu L, Lin Z, Yu Z, et al. Orientin downregulating oxidative stress-mediated Endoplasmic reticulum stress and mitochondrial dysfunction through AMPK/SIRT1 pathway in rat nucleus pulposus cells in vitro and attenuated intervertebral disc degeneration in vivo. Apoptosis. 2022;27(11–12):1031–48. 36125665 10.1007/s10495-022-01770-9

[24] Basatvat S, Bach FC, Barcellona MN, Binch AL, Buckley CT, Bueno B, Chahine NO, Chee A, Creemers LB, Dudli S, et al. Harmonization and standardization of nucleus pulposus cell extraction and culture methods. JOR Spine. 2023;6(1):e1238. 36994456 10.1002/jsp2.1238 PMC10041384

[25] Zhang J, Li Z, Chen F, Liu H, Wang H, Li X, Liu X, Wang J, Zheng Z. TGF-β1 suppresses CCL3/4 expression through the ERK signaling pathway and inhibits intervertebral disc degeneration and inflammation-related pain in a rat model. Exp Mol Med. 2017;49(9):e379. 28935976 10.1038/emm.2017.136 PMC5628275

[26] Yang H, Gao F, Li X, Wang J, Liu H, Zheng Z. TGF-β1 antagonizes TNF-α induced up-regulation of matrix metalloproteinase 3 in nucleus pulposus cells: role of the ERK1/2 pathway. Connect Tissue Res. 2015;56:461–8. 26075533 10.3109/03008207.2015.1054030

[27] Gruber HE, Norton HJ, Hanley EN Jr. Anti-apoptotic effects of IGF-1 and PDGF on human intervertebral disc cells in vitro. Spine (Phila Pa 1976). 2000;25(17):2153–7. 10973395 10.1097/00007632-200009010-00002

[28] Séguin CA, Bojarski M, Pilliar RM, Roughley PJ, Kandel RA. Differential regulation of matrix degrading enzymes in a TNFalpha-induced model of nucleus pulposus tissue degeneration. Matrix Biol. 2006;25(7):409–18. 16934445 10.1016/j.matbio.2006.07.002

[29] Seki S, Asanuma-Abe Y, Masuda K, Kawaguchi Y, Asanuma K, Muehleman C, Iwai A, Kimura T. Effect of small interference RNA (siRNA) for ADAMTS5 on intervertebral disc degeneration in the rabbit anular needle-puncture model. Arthritis Res Ther. 2009;11(6):R166. 19889209 10.1186/ar2851 PMC3003501

[30] Liu S, Hu Y, Xu W, Liu W, Wang B, Zeng X, Shao Z, Yang C, Xiong L, Cai X. Restoration of lysosomal function attenuates autophagic flux impairment in nucleus pulposus cells and protects against mechanical overloading-induced intervertebral disc degeneration. Autophagy. 2025;21(5):979–95. 39675125 10.1080/15548627.2024.2440844 PMC12013417

[31] Ye F, Xu Y, Lin F, Zheng Z. TNF-α suppresses SHOX2 expression via NF-κB signaling pathway and promotes intervertebral disc degeneration and related pain in a rat model. J Orthop Res. 2021;39(8):1745–54. 32816304 10.1002/jor.24832

[32] Zhang W, Gong Y, Zheng X, Qiu J, Jiang T, Chen L, Lu F, Wu X, Cheng F, Hong Z. Platelet-Derived growth Factor-BB inhibits intervertebral disc degeneration via suppressing pyroptosis and activating the MAPK signaling pathway. Front Pharmacol. 2021;12:799130. 35095507 10.3389/fphar.2021.799130 PMC8795915

[33] Li B, Zheng XF, Ni BB, Yang YH, Jiang SD, Lu H, Jiang LS. Reduced expression of insulin-like growth factor 1 receptor leads to accelerated intervertebral disc degeneration in mice. Int J Immunopathol Pharmacol. 2013;26(2):337–47. 23755749 10.1177/039463201302600207

[34] Wu QJ, Zhang TN, Chen HH, Yu XF, Lv JL, Liu YY, Liu YS, Zheng G, Zhao JQ, Wei YF, et al. The Sirtuin family in health and disease. Signal Transduct Target Ther. 2022;7(1):402. 36581622 10.1038/s41392-022-01257-8 PMC9797940

[35] Liu S, Wu N, Liu J, Liu H, Su X, Liu Z, Zuo Y, Chen W, Liu G, Chen Y, et al. Association between ADAMTS-4 gene polymorphism and lumbar disc degeneration in Chinese Han population. J Orthop Res. 2016;34(5):860–4. 26495885 10.1002/jor.23081

[36] Liu C, Yang H, Gao F, Li X, An Y, Wang J, Jin A. Resistin promotes intervertebral disc degeneration by upregulation of ADAMTS-5 through p38 MAPK signaling pathway. Spine (Phila Pa 1976). 2016;41(18):1414–20. 26974833 10.1097/BRS.0000000000001556

[37] Rubinsztein DC, Shpilka T, Elazar Z. Mechanisms of autophagosome biogenesis. Curr Biol. 2012;22(1):R29–34. 22240478 10.1016/j.cub.2011.11.034

[38] Chao CC, Huang CL, Cheng JJ, Chiou CT, Lee IJ, Yang YC, Hsu TH, Yei CE, Lin PY, Chen JJ, et al. SRT1720 as an SIRT1 activator for alleviating paraquat-induced models of parkinson’s disease. Redox Biol. 2022;58:102534. 36379180 10.1016/j.redox.2022.102534 PMC9663539

[39] Dai H, Sinclair DA, Ellis JL, Steegborn C. Sirtuin activators and inhibitors: promises, achievements, and challenges. Pharmacol Ther. 2018;188:140–54. 29577959 10.1016/j.pharmthera.2018.03.004 PMC6342514

[40] Chen F, Liu H, Wang X, Li Z, Zhang J, Pei Y, Zheng Z, Wang J. Melatonin activates autophagy via the NF-κB signaling pathway to prevent extracellular matrix degeneration in intervertebral disc. Osteoarthritis Cartilage. 2020;28(8):1121–32. 32470597 10.1016/j.joca.2020.05.011

[41] Zhao K, Zhang Y, Kang L, Song Y, Wang K, Li S, Wu X, Hua W, Shao Z, Yang S, et al. Methylation of microRNA-129-5P modulates nucleus pulposus cell autophagy by targeting Beclin-1 in intervertebral disc degeneration. Oncotarget. 2017;8(49):86264–76. 29156793 10.18632/oncotarget.21137 PMC5689683

[42] Sun K, Jing X, Guo J, Yao X, Guo F. Mitophagy in degenerative joint diseases. Autophagy. 2021;17(9):2082–92. 32967533 10.1080/15548627.2020.1822097 PMC8496714

[43] Song Y, Lu S, Geng W, Feng X, Luo R, Li G, Yang C. Mitochondrial quality control in intervertebral disc degeneration. Exp Mol Med. 2021;53(7):1124–33. 34272472 10.1038/s12276-021-00650-7 PMC8333068

[44] Saberi M, Zhang X, Mobasheri A. Targeting mitochondrial dysfunction with small molecules in intervertebral disc aging and degeneration. Geroscience. 2021;43(2):517–37. 33634362 10.1007/s11357-021-00341-1 PMC8110620

[45] Qi Y, Tang R, Shi Z, Feng G, Zhang W. Wnt5a/Platelet-rich plasma synergistically inhibits IL-1β-induced inflammatory activity through NF-κB signaling pathway and prevents cartilage damage and promotes meniscus regeneration. J Tissue Eng Regen Med. 2021;15(7):612–24. 33843153 10.1002/term.3198

[46] Weng Y, Zhang W, Qu F, Deng Z, Zhang X, Liu S, Wei H, Hao T, Gao L, Zhang M et al. Human platelet-rich plasma promotes primordial follicle activation via the PI3K/Akt signaling pathway. Mol Hum Reprod 2025, 31(2). 10.1093/molehr/gaaf007 40088933

[47] Williams RJ, Laagland LT, Bach FC, Ward L, Chan W, Tam V, Medzikovic A, Basatvat S, Paillat L, Vedrenne N, et al. Recommendations for intervertebral disc notochordal cell investigation: from isolation to characterization. JOR Spine. 2023;6(3):e1272. 37780826 10.1002/jsp2.1272 PMC10540834

